# A case of blunt thoracic aortic injury requiring ECMO for acute malperfusion before TEVAR

**DOI:** 10.1186/s13049-017-0456-z

**Published:** 2017-11-22

**Authors:** Daiki Wada, Koichi Hayakawa, Shuji Kanayama, Shuhei Maruyama, Hiromu Iwamura, Noriyuki Miyama, Fukuki Saito, Yasushi Nakamori, Yasuyuki Kuwagata

**Affiliations:** 1 Department of Emergency and Critical Care Medicine, Kansai Medical University Medical Center, 10-15 Fumizono-cho, Moriguchi, Osaka, 570-8507 Japan; 2 Department of Vascular Surgery, Kansai Medical University Medical Center, 10-15 Fumizono-cho, Moriguchi, Osaka, 570-8507 Japan; 3 Department of Emergency and Critical Care Medicine, Kansai Medical University Hospital, 2-3-1 Shinmachi, Hirakata, Osaka, 573-1191 Japan

**Keywords:** Thoracic endovascular aneurysm repair (TEVAR), Veno-arterial extracorporeal membrane oxygenation (VA ECMO), Blunt thoracic aortic injury (BTAI)

## Abstract

**Background:**

Blunt thoracic aortic injury (BTAI) is associated with a high mortality rate and the paradigm of treating patients with BTAI currently favours thoracic endovascular aneurysm repair (TEVAR) if possible. In BTAI, lethal malperfusion caused by a pseudoaneurysm has rarely been reported. We present the first report of a successful case in which a pseudoaneurysm causing the infrequent occurrence of lethal malperfusion and subsequent acute severe ischaemia in the distal portion of the thoracic descending aorta was overcome by veno-arterial extracorporeal membrane oxygenation (VA ECMO) as a bridging therapy until the initiation of TEVAR.

**Case presentation:**

An adult woman was transferred to our emergency room after injuries sustained by falling from height. Her vital signs were unstable on admission. CT examination revealed the multiple injuries: traumatic subarachnoid haemorrhage, severe unstable pelvic fracture, and a grade III injury of the thoracic aorta. We made the decision to perform TEVAR after external fixation and transcatheter arterial embolization (TAE) for the pelvic injury. During preparations for TEVAR, her lower limbs rapidly felt cold, and her blood lactate level and serum potassium rapidly increased. By the clinical data and ultrasonography and lower extremity Doppler, we diagnosed severe ischaemia in distal portion of the descending aorta caused by a pseudoaneurysm proximal to the descending thoracic aorta. Because we still had not prepared for TEVAR, we immediately started VA ECMO until TEVAR could begin. After the initiation of VA ECMO, her lactate and potassium levels could be controlled. Under VA ECMO support, she underwent TEVAR. After inpatient rehabilitation, she was discharged home without neurologic sequelae.

**Conclusions:**

VA ECMO could be an important, less-invasive treatment as a bridging therapy for acute severe malperfusion syndrome until TEVAR is initiated for BTAI.

## Background

Blunt thoracic aortic injury (BTAI) is a life-threatening event that is most commonly associated with deceleration injuries [1]. Up to 75% of deaths caused by blunt trauma are secondary to chest injuries, with the majority of these deaths arising in patients with BTAI [2]. With the advent of a minimally invasive approach to the aorta via thoracic endovascular aneurysm repair (TEVAR), TEVAR has rapidly become a standard of care in the treatment of these injuries, negating the need for an open thoracotomy, aortic cross-clamping, anticoagulation, or cardiopulmonary bypass [3]. We report a case of successful TEVAR in a critically ill patient with multiple injuries. During preparations for TEVAR, acute severe ischaemia developed in the distal portion of the descending thoracic aorta, and this lethal malperfusion was overcome by veno-arterial extracorporeal membrane oxygenation (VA ECMO) as a bridging therapy until TEVAR could be initiated. To our knowledge, this is the first case report of BTAI in which acute severe ischemia in the distal descending thoracic aorta was treated with VA ECMO bridging therapy.

## Case presentation

**Fig. 1:**
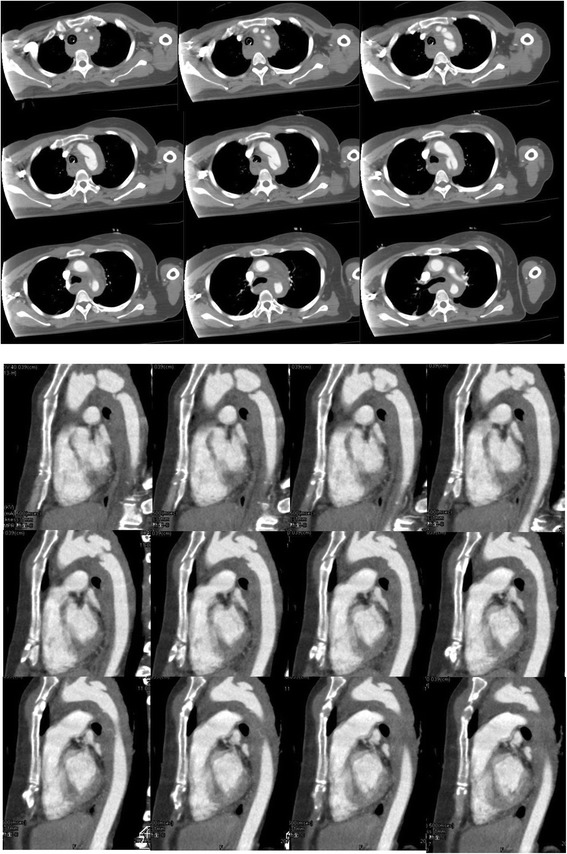
Enhanced CT on admission showed a grade III injury of the thoracic aorta on both axial and sagittal imaging

**Fig. 2:**
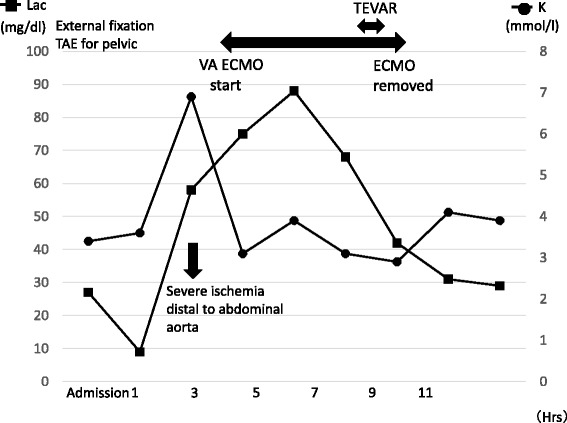
Clinical course of this case. Lac (mg/dl): lactate level; K (mmol/l): serum potassium level; TEVAR: thoracic endovascular aortic repair; VA ECMO: veno-arterial extracorporeal membrane oxygenation

**Fig. 3:**
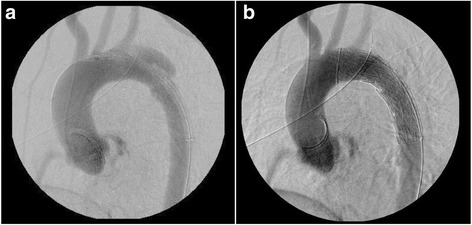
**a**: Aortogram before deployment of stent showed the traumatic thoracic pseudoaneurysm. **b**: Post-stent deployment aortogram showed no pooling of contrast and a patent left subclavian artery

**Fig. 4:**
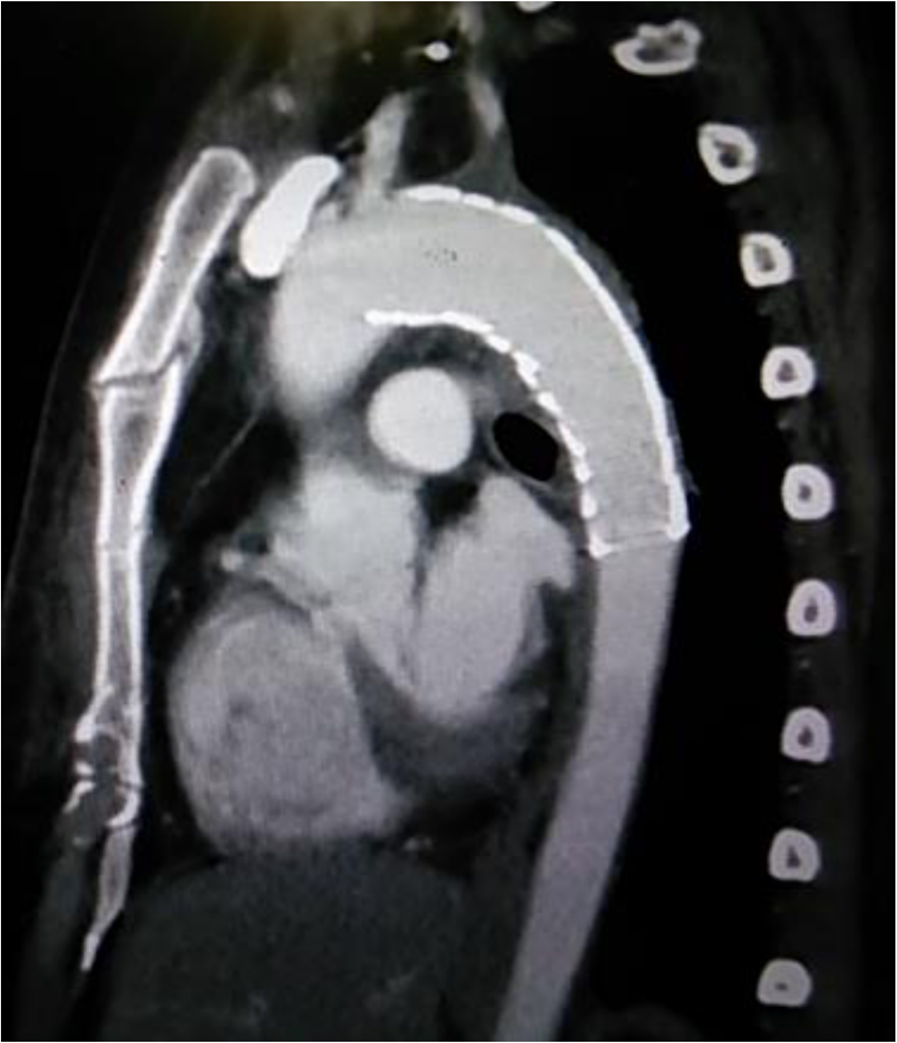
CT image after stent grafting showed no evidence of endoleak, and the stent was in a good position

An adult woman was transferred to our emergency room after injuries sustained by falling from height. Her Glasgow Coma Scale on arrival was 10/15, and her vital signs were unstable on admission. She was intubated and fluid administration was started immediately. A chest tube was inserted for a pneumothorax. Computed tomography (CT) examination revealed the following injuries: traumatic subarachnoid haemorrhage, multiple bilateral traumatic rib fractures, severe unstable pelvic fracture, fracture of the right tibia, fracture of the right humerus, and a grade III injury of the thoracic aorta (Fig. 1). CT angiography confirmed a dissection of the descending thoracic aorta just proximal to the origin of the left subclavian artery. Her injury severity score (ISS) was 59, Revised Trauma Score (RTS) was 5.15, and the probability of survival (Ps) by the Trauma and Injury Severity Score (TRISS) method was 29.67. A multidisciplinary trauma team initiated definitive intervention. She was at very high risk for intervention for BTAI in consideration of her traumatic subarachnoid and pelvic haemorrhages. First, we performed external fixation and transcatheter arterial embolisation (TAE) for the pelvic injury. Then, we confirmed no critical increase in subarachnoid haemorrhage by another CT examination. We made the decision to perform TEVAR. The patient’s blood lactate level was 35 mg/dl at admission, and it had decreased to 9 mg/dl following fluid resuscitation and external fixation and TAE for pelvic injury. During the TAE, angiography of the abdominal aorta showed poor blood flow. About 3 h after admission, both of her lower limbs felt cold, and her blood lactate level rapidly rose to 58 mg/dl, and serum potassium rapidly increased to 7 mmol/L (Fig. 2). We diagnosed severe ischaemia in distal portion of the descending aorta caused by a pseudoaneurysm proximal to the descending thoracic aorta because abdominal ultrasonography revealed no flow in the abdominal aorta, and lower extremity Doppler showed no flow in either lower extremity. Because we still had not prepared for TEVAR, we immediately started VA ECMO to improve the severe malperfusion in her lower body until TEVAR could begin. In VA ECMO, a venous cannula was placed in the right common femoral vein for extraction and an arterial cannula was placed into the right femoral artery for infusion. After the initiation of VA ECMO, her lactate and potassium levels could be controlled. Under VA ECMO support and general anaesthesia, she underwent TEVAR by the vascular team 8 h after admission. Access for the delivery device was via her right femoral artery. A GORE TAG device (W. L. Gore & Associates, Flagstaff, AZ) was deployed just proximal to the origin of the left subclavian artery (Fig. 3). The patient tolerated the procedure well, and there were no complications. The VA ECMO cannulas were removed on post-trauma day 1. A CT scan performed on post-trauma day 30 showed no evidence of endoleak, and the stent was in a good position (Fig. 4). On post-trauma day 7, her severe pelvic fracture was treated with transiliac and iliosacral screws. After inpatient rehabilitation, she was discharged home on post-trauma day 127 without neurologic sequelae.

## Discussion

BTAI is associated with a high mortality rate and has been implicated as the second most common cause of death in trauma patients after intracranial haemorrhage [4, 5]. It has been estimated that less than 25% of patients with such an injury live to be evaluated in a hospital [6], and of those who do, up to 50% will die within 24 h [7]. Open repair for BTAI is associated with high rates of morbidity and mortality, particularly in trauma patients with multiple injuries. The first reported case of TEVAR was reported in 1997 [8], and in the following decades, this technique has become the most commonly used treatment for BTAI. TEVAR offers the potential for a durable aortic repair while avoiding the morbidity associated with thoracotomy, aortic cross-clamping, and cardiopulmonary bypass [9].

The continued advancement of endovascular technologies, including stents specific for aortic trauma, and operator experience has decreased the adverse events of endoleak after TEVAR [10]. Paralysis, stroke, and left upper extremity ischaemia with left subclavian artery coverage have also significantly decreased over time [6, 9, 11, 12]. TEVAR is now a clear trend favouring a minimally invasive approach over surgical repair. The frequent use of stents may lead to decreases in operation time and blood loss and substitution of packed red cells as well as a reduction in manpower [13]. However, long-term outcomes of TEVAR in trauma patients are largely unknown. Brenner et al. suggested from their long-term outcome data that TEVAR is a feasible treatment modality for BTAI, and it may be at least comparable to open repair [14]. In a comprehensive meta-analysis review, Murad et al. reported the mortality rates of patients who were treated with TEVAR, open repair, and nonoperative medical management to be 9%, 19%, and 46%, respectively (*p* < 0.01) [15]. The current Society for Vascular Surgery Clinical Practice Guidelines suggest urgent (<24 h) thoracic endovascular aortic repair for Grade II to Grade IV BTAI [9].

In our case, we infrequently experience acute severe ischaemia in the distal portion of the descending aorta caused by a pseudoaneurysm proximal to the descending thoracic aorta. Using BTAI patient data from a multicentre trial, Khoynezhad et al. reported that an aortic injury-related cause of early death was haemothorax [16]. Brenner et al. reported in the long-term outcomes of TEVAR that overall mortality was due to sepsis, cardiac arrest, liver injury, and traumatic brain injury [14]. Approximately 25% of patients presenting with acute type B aortic dissection are complicated at admission by malperfusion syndrome or haemodynamic instability, resulting in a high risk of early death if untreated [17–19]. Malperfusion syndrome is the most frequent complication of type B dissection [20]. Currently, the less invasive method of endovascular repair provides a better 30-day/in-hospital survival for complicated acute type B aortic dissection [20]. In BTAI, lethal malperfusion caused by a pseudoaneurysm has rarely been reported.

Our emergency department cannot always perform emergency TEVAR because the operators skilled in TEVAR and the emergency operation room are not always available. This problem of logistics was experienced in the present case, and we had to wait for about 8 h until we could complete preparations for TEVAR. In view of the multiple trauma suffered by our patient, we decided to perform VA ECMO as a less invasive method for severe malperfusion syndrome until TEVAR could be initiated. ECMO has been reported in the management of patients with aortic injuries [21], but cases of BTAI requiring ECMO until TEVAR or during TEVAR have rarely been reported. Lee et al. reported a case of the emergent rescue application of ECMO in a patient with profound cardiac and respiratory failure during aortic stent-graft repair [22]. To our knowledge, this is the first report of a successful case in which a pseudoaneurysm causing the infrequent occurrence of lethal malperfusion and subsequent acute severe ischaemia in the distal portion of the thoracic descending aorta was overcome by VA ECMO as a bridging therapy until the initiation of TEVAR. In the trauma centres in which TEVAR cannot always be performed emergently, as in our hospital, we think that VA ECMO could be an important, less-invasive treatment for acute severe malperfusion syndrome until TEVAR can be initiated for BTAI.

## Conclusion

During preparations for TEVAR, a pseudoaneurysm caused the infrequent occurrence of acute severe ischaemia in the distal portion of the descending thoracic aorta, and the malperfusion syndrome was successfully overcome by VA ECMO as a bridging therapy until TEVAR could be initiated.

## Abbreviations

BTAI: Blunt thoracic aortic injury
ECMO: Extracorporeal membrane oxygenation
TEVAR: Thoracic endovascular aortic repair
VA: Veno-arterial
